# ParaMED Home: A protocol for a randomised controlled trial of paramedic assessment and referral to access medical care at home

**DOI:** 10.1186/1471-227X-11-7

**Published:** 2011-06-08

**Authors:** Glenn Arendts, Moira Sim, Steven Johnston, Richard Brightwell

**Affiliations:** 1 Centre for Clinical Research in Emergency Medicine, Western Australian Institute for Medical Research, Australia; 2 School of Primary Aboriginal and Rural Health Care, University of Western Australia, Australia; 3 Systems Intervention Research Centre for Health, Edith Cowan University, Australia

## Abstract

**Abstract:**

**Background**

In Australia approximately 25% of Emergency Department (ED) attendances are via ambulance. ED overcrowding in Australia, as in many countries, is common. Measures to reduce overcrowding include the provision of enhanced timely primary care in the community for appropriate low risk injury and illness. Therefore paramedic assessment and referral to a community home hospital service, in preference to transfer to ED, may confer clinical and cost benefit.

**Methods/Design**

A randomised controlled trial. Consenting adult patients that call an ambulance and are assessed by paramedics as having an eligible low risk problem will be randomised to referral to ED via ambulance transfer or referral to a rapid response service that will assess and treat the patient in their own residence. The primary outcome measure is requirement for unplanned medical attention (in or out of hospital) in the first 48 hours. Secondary outcomes will include a number of other clinical endpoints. A cost effectiveness analysis will be conducted.

**Discussion**

If this trial demonstrates clinical non-inferiority and cost savings associated with the primary assessment service, it will provide one means to safely address ED overcrowding.

**Trial Registration:**

Australian and New Zealand Clinical Trials Registry Number 12610001064099

## Background

Emergency Department (ED) overcrowding is an international phenomenon[1]. Overcrowding is associated with mortality[2], delay to time critical therapy[3,4], patient dissatisfaction[5] and ambulance ramping, where paramedics are unable to deliver patients to ED due to a lack of available beds.

In Australia, there are over 7 million hospital ED episodes of care per year with up to 25% of patients arriving in ED by ambulance[6]. Estimates of the proportion of these ED cases that are primary care patients vary according to the definition and scope of primary care[7], but a reasonable proportion of cases that present to ED by ambulance may be equally suited to care in the community by a primary care service.

There are many reasons why people call an ambulance in addition to a medical emergency. There may be inability to access alternative health care; issues associated with chronic illness and disability; requirements for advice and reassurance; and psychosocial reasons. Many patients access health care through the ambulance service without necessarily believing they need transport to hospital, yet paramedics in some jurisdictions are governed by the duty of care that requires them to render assistance and care for the patient until they handover to another health service. In practice this means that the patient may be transferred to ED even if the patient does not require emergency care.

There is evidence from cluster and before-after studies that paramedics with extended skills can mange low-risk patients in the community without hospital transfer[8,9]. However other studies using paramedics as an assessment and referral service to divert cases away from ED have yielded conflicting results[10]. This trial has been designed to provide an alternative care path for patients that call an ambulance for minor injury and illness. The aim of the trial is to determine whether paramedic referral to a rapid response primary care service in the patient's own residence is clinically effective, safe and an efficient use of resources.

## Methods/Design

### Ethical considerations

The study has received human research ethical approval from Edith Cowan University (5249 SIM), the Department of Health Western Australia (#2010/45) and the Silver Chain Association of Western Australia (EC App 066).

### Study setting

The study will commence in 2011 and be conducted in metropolitan Perth, Western Australia - a city with a population of over one million people serviced mainly by a single not-for-profit ambulance service (St John Ambulance Australia WA Inc) contracted by the government. The ambulance service transport adult patients to one of three tertiary or four district ED within the metropolitan area. Currently there are in excess of 100000 ambulance transfers to these ED annually.

### Study design

A randomised controlled trial, with consenting eligible patients randomised to either the intervention or usual care (ED transfer).

### Eligibility criteria

#### a. Inclusion

To be eligible for the trial patients will have been attended by paramedics in their own residence for *any *of the following suspected clinical conditions

1. Isolated minor injury from low risk mechanism e.g. simple laceration, isolated distal limb injury, simple contusion

2. Simple infection e.g. below knee cellulitis, influenza-like illness

3. Hardware problem e.g. blocked or displaced bladder catheter

#### b. Exclusion

Patients will be excluded if they meet *any *of the following criteria

1. Age < 16 years

2. Third trimester pregnancy

3. Not in own residence when attended by paramedics

4. Residence is unsafe environment for patient (e.g. living alone and requiring supervision) or staff

5. Glasgow Coma Score < 15

6. SaO_2 _< 95% in room air

7. Heart rate > 100/min

8. Systolic BP < 100 mm Hg

9. Severe pain requiring narcotic analgesia

10. Paramedics assess patient as being unsuitable to wait up to four hours for assessment and management

### Consent and enrolment procedures

Patients will be enrolled by paramedics that have been trained in the research protocol. Eligible patients will be identified by the paramedics using a checklist, and patients will be provided with verbal and written information in an Information and Consent Form. Written consent will incorporate agreement to being randomised to the intervention or control arm, to allow access to medical records and to allow a follow up telephone call at 28 days post enrolment. If patients do not consent to the study they will be transported to hospital as per usual practice. Patients may choose to withdraw consent at any time without prejudice. Patients randomised to the intervention arm will be advised to make a second call for paramedic assistance if they feel their condition has worsened prior to the arrival of the home hospital team.

Upon attainment of written consent, paramedics will call the central ambulance emergency call centre to confirm suitability for the trial with a Clinical Support Officer, provide information about the enrolment and obtain trial allocation. Patients will be randomised through a computer generated randomisation process at the call centre, and the paramedics, at the scene, informed to either refer the patient by telephone to the priority response home hospital service (intervention arm) or transport the patient to ED (control arm).

### Intervention

The intervention arm will be a priority response home hospital service run by the Silver Chain Association of Western Australia that will visit the patient in their own residence within four hours of paramedic referral. A nurse practitioner or clinical nurse specialist will provide the initial episode of care with 24 hour medical cover provided by an on-call roster of general practioners and specialists. Assessment, investigation and treatment as required will occur in the community. After the initial episode of care in their residence the patient may be discharged from the care of the service; have ongoing therapy at home over ensuing days e.g. IV antibiotics; or if needed referred to an ED.

### Control

The control is transfer of the patient to a public hospital ED for medical assessment, investigation and treatment as required.

### Outcome measures

#### a. Primary

The primary outcome measure is the proportion of patients requiring one or more episodes of unplanned medical attention (in or out of hospital) in the first 48 hours.

#### b. Secondary

1. Proportion of patients requiring 2nd ambulance attendance prior to the arrival of the primary care team.

2. Number of ambulance transports of patients to ED following referral to the intervention.

3. Proportion of patients referred to ED in the control arm that did not wait for assessment/treatment

4. Time to first contact with definitive care provider (ED and home hospital team).

5. Number of investigations performed

6. Number of calls to ambulance call centre within 48 hours.

7. Number of episodes of contact with healthcare providers in next 7 days.

8. Number of episodes of ED attendance within the next 7 days.

9. Hospital admissions within the next 7 days.

10. Adverse events within the next 7 days.

11. Deaths within the next 7 days.

#### c. Cost benefit

A cost benefit analysis will be performed to reveal economic outcomes. Difference between the annualised extra fixed and variable costs and the annualised savings in health system costs will determine the net benefit, if any, of the intervention.

Costs will be calculated as fixed costs (e.g. equipment costs depreciated over the life of the equipment giving annual costs) and variable costs (e.g. labour costs for the Home Hospital program service and paramedic training costs). Benefits will be calculated through differences between the intervention and control groups in ambulance usage, hospital separations and ED visits.

#### d. Patient satisfaction

Patient satisfaction with whichever arm they were randomised to will be measured at 28 day telephone follow up using a series of rating scale questions. Participants will be asked to report on satisfaction with the timeliness of the services, the explanation received, the care received, convenience of the service, follow up arrangements, staff attitudes and overall service. They will also be asked about their perception of safety within the service, perception of adequate diagnosis and symptom relief, and their preference for treatment at hospital or home hospital.

### Data collection mechanisms

Initial enrolment data will be collected using preformatted data sheets to be completed by paramedic staff. Once randomised, patients will be followed up by research staff employed specifically for the project. Staff will use electronic hospital databases, supplemented by individual patient medical records where necessary, to determine resource utilisation and outcome data for patients randomised to the comparator arm. Staff will use existing databases to collect similar data on patients in the intervention arm. 28 day follow up will be by telephone call supplemented by data from the data linkage unit at the Health Department of Western Australia.

### Sample size estimates

Based on an estimate of 5% of patients having an unplanned episode of care within 48 hours, to detect non-inferiority defined as a risk ratio no greater than 1.5 between the intervention and control groups a sample size of 940 patients in each group will be required (if α = 0.05 and β= 0.2). We estimate 10% of the 100000 annual ambulance transfers will be eligible for the trial so anticipate being able to recruit the requisite sample size in less than one year.

### Statistical plan

We will use summary descriptive statistics for the study population. Dichotomous outcomes will be compared between the intervention and comparator groups using Pearson's chi square test and by calculating relative risk. Continuous outcomes will be compared using an independent t test for normally distributed data and Mann Whitney U test for non-parametric data. Cost benefit will be expressed in dollar terms. Patient satisfaction outcomes will be largely descriptive.

## Discussion

This trial is a methodologically rigorous and adequately powered evaluation of an alternative to hospital transfer for low acuity patients calling an ambulance service. There are large potential healthcare benefits in terms of reducing ED overcrowding, increasing paramedic availability, reducing ambulance ramping, cost savings and improving patient satisfaction. The study outcomes will provide important information on the safety and quality of assessment and care in the home for conditions which would otherwise result in a presentation to ED.

This is the first time that paramedics will be involved in a trial in which their assessment determines suitability for handover to a service outside of an ED. The clinical processes and pathways established in the trial will therefore provide evaluated practice as a basis for future health policy.

The trial has one major risk - inferior clinical outcomes associated with either misdiagnosis or inadequate therapy in the intervention arm. Although both paramedic and nurse practitioners are highly experienced health professionals and will have immediate access to specialist and generalist medical practitioners, there will be no medical diagnostician in the direct care of the vast majority of intervention arm patients. However we anticipate with careful patient inclusion and exclusion criteria and well trained staff, that we can select a sufficiently low risk study population and show clinical non-inferiority between the groups. We anticipate benefits to lie in cost savings and patient satisfaction.

## Competing interests

GA serves as a medical advisor to St John Ambulance Australia WA Inc and the Silver Chain Association of Western Australia. He receives no financial benefit from the participation of either organisation in this research.

## Authors' contributions

All authors have contributed to design of the trial protocol. MS has overall responsibility for trial governance. GA wrote the manuscript that was edited by all authors. All authors have read and approved the final manuscript.

## Pre-publication history

The pre-publication history for this paper can be accessed here:

http://www.biomedcentral.com/1471-227X/11/7/prepub
